# Critical Rolling Process Parameters for Dynamic Recrystallization Behavior of AZ31 Magnesium Alloy Sheets

**DOI:** 10.3390/ma11102019

**Published:** 2018-10-18

**Authors:** Wenke Wang, Qing Miao, Xuemin Chen, Yang Yu, Wencong Zhang, Wenzhen Chen, Erde Wang

**Affiliations:** 1 School of Materials Science and Engineering, Harbin Institute of Technology, Weihai 264209, China; 15B309020@hit.edu.cn (W.W.); CXMClaire0218@126.com (X.C.); hityyang@hit.edu.cn (Y.Y.); zwinc@hitwh.edu.cn (W.Z.); nclwens@hit.edu.cn (W.C.); mujie_02@163.com (E.W.); 2 School of Materials Science, Shanghai Dian Ji University, Shanghai 201306, China

**Keywords:** AZ31, dynamic recrystallization, process parameter, texture

## Abstract

In this work, the influence of various rolling temperatures and thickness reductions on the dynamic recrystallization (DRX) behavior of AZ31 magnesium alloy sheets was investigated. Meanwhile, the texture variation controlled by DRX behavior was analyzed. Results suggested that, with the help of DRX behavior, reasonable matching of rolling temperature and thickness reduction could effectively refine the grain size and improve the microstructure homogeneity. Using the grain refinement and microstructure homogeneity as the reference, the critical rolling process parameters were 400 °C—30%, 300 °C—30%, and 250 °C—40% in the present work. In terms of basal texture variation, the occurrence of twins produced the largest maximum texture intensity. However, for the sheets with DRX behavior, the maximum texture intensity decreased sharply, but would steadily increase with the growth of DRXed grain. Additionally, for DRXed grains, the <11-20>//RD (RD: rolling direction) grains would gradually annex the <10-10>//RD grains with the growth of DRXed grains, which finally made their texture component become the dominant texture state. However, when the deformation continued, the <10-10> in DRXed grains would rotate toward the RD again. Weighted by the fracture elongation of AZ31 magnesium alloy sheet, the critical thickness reductions were 30–40% under the rolling temperature of 400 °C.

## 1. Introduction

Magnesium alloys are recognized as the lightest structure metal and, due to their high specific strength and stiffness, they have received significant attention in many fields, such as electronics, automotive, and aerospace [1,2]. However, owing to their inherent hexagonal close packed (HCP) structure, only two independent basal slips can be activated at room temperature, which leads to their poor low temperature formability and, finally, restricts their wide use in critical safety components [3,4].

Grain refinement was an effective way to improve the room temperature formability of magnesium alloys [5,6,7]. Many literature studies have shown that microalloying was an effective way to realize grain refinement according to a particle-stimulated nucleation mechanism (PSN) [6,8]. However, the high cost and limited supply of rare earth elements made magnesium alloys prohibitive in many applications [9]. Nowadays, various plastic process techniques could also effectively refine the grain size based on dynamic recrystallization (DRX) behavior, such as hot rolling (HR) [10,11], twin roll casting (TRC) [12], equal channel angular rolling (ECAR) [13], and different speed rolling (DSR) [14]. In view of this, Kim et al. applied asymmetrical rolling to fabricate AZ31 magnesium alloy sheets with a fine grain size 1.4 μm and its maximum elongation could achieve 35% [15]. Cho et al. compared the deep drawing of ZK60 magnesium sheets fabricated using ingot and TRC methods, and it was found that the TRC samples possessed a smaller grain size and higher elongation of 18% [12]. However, in the above process methods, hot rolling was considered to be the most suitable method for industrial sheet fabrication, owing to the absence of intermediate annealing. Prior work had suggested that hot rolling could not only refine the grain size, but also improve the microstructure homogeneity via multiple DRX [5,16]. Essentially, DRXed grain size was closely related to the Zener–Hollomon parameter *Z* ($Z=\dot{\varepsilon }\;\exp(Q/RT)$, where $\dot{\varepsilon }$ is the strain rate, *T* is the deformation temperature, *Q* is the activation energy, and *R* is the gas constant) [16,17]. This meant that grain refinement could be realized by carefully controlling *Z* during plastic deformation. Recently, Liu et al. studied the microstructure of as-rolled AZ31 magnesium alloy sheets with various thickness reductions from 10% to 60%, under similar lower rolling temperature, and pointed out that the 10% sheet consisted of deformed grains, while the 60% sheet completed DRXed grains [18]. This demonstrated that, on the premise of no cracking in the sheets, a lower rolling temperature could also realize DRX if the deformation was large enough as well and, in this case, grain refinement tended to be more obvious [5]. The above result also suggested that DRX behavior was a comprehensive outcome of many factors, instead of depending on a single factor. If this internal mechanism of DRX could be well understood under different process parameters, it would help to obtain a finer and homogeneous microstructure and, finally, improve the properties of magnesium alloys. However, to the authors’ knowledge, DRX behavior under different process parameters has not been investigated systematically, especially in the hot rolling process. Importantly, many studies pointed out that, in magnesium alloys, the occurrence of DRX would cause texture variations which could further influence their mechanical properties [8,9,19]. In terms of the formability of magnesium alloy sheets, a weak basal texture was generally conducive to improve their formability [20,21]. For example, Huang et al. adopted a combination process of high-temperature rolling and annealing to fabricate AZ31 magnesium alloy sheets, and found that their Erichsen values were significantly increased, from 4.5 mm to 8.6 mm, owing to the decreased texture intensity from 5.4 to 2.7 [22]. This further urged the necessity of exploiting DRX behavior under different rolling process parameters. Therefore, in order to find the critical rolling process parameters which realized the fabrication of AZ31 magnesium alloy sheets with fine and homogeneous microstructure, DRX behavior was investigated under different rolling process parameters. Meanwhile, the influence of DRX behavior on texture variation during hot rolling process was analyzed in detail.

## 2. Experimental Procedures

**Figure 1:**
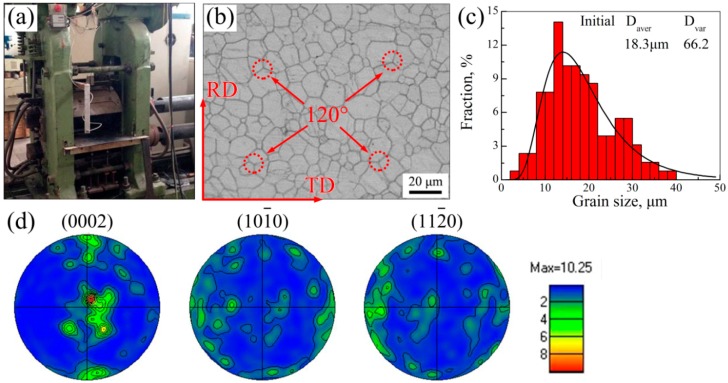
The equipment of the rolling mill (**a**), and microstructure characteristics regarding metallography observation (**b**), corresponding grain size distribution (**c**), and (0002), (10-10), (11-20) pole figures of initial material (**d**) (RD: rolling direction, TD: transverse direction).

Hot-rolled AZ31 magnesium alloy sheets with 4 mm in thickness were used as the initial material in this work. The nominal composition of this initial material was Mg-3wt.% Al-1wt.% Zn-0.2wt.% Mn. As previously described, Zener–Hollomon parameter *Z* was closely related to the strain rate and rolling temperature. According to the literature [23], the rolling strain rate could be calculated by $\dot{\varepsilon }=\frac{H-h}{H}\frac{v}{\sqrt{r\left(H-h\right)}}$ (*H* is the initial thickness, *h* is the thickness after hot rolling, *ν* is the rolling speed, and *r* is the semi-diameter of the roller). Clearly, *h* was directly affected by thickness reduction. Moreover, *H*, *ν*, and *r* were deemed to be constants in this work. Thus, in the present work, thickness reduction and rolling temperature were the variables which influenced DRX behavior. Based on this, the initial materials were rolled in a single pass with thickness reductions of 20%, 30%, 40%, and 50% under the rolling temperatures of 400 °C, 300 °C, 250 °C, and 200 °C, respectively. Moreover, in order to improve the workability of initial material, all sheets were preheated to 400 °C for 30 min prior hot rolling. Hot rolling was performed on a two-high mill with 110 mm in roll semi-diameter at a roll velocity of 5 m/min. The main rolling parameters, including rolling temperature and thickness reduction, are summarized in Table 1. Figure 1 shows the equipment of the rolling mill and the microstructure characteristics of the initial material.

As shown in Figure 1b, the microstructure in initial material consisted of equiaxed grains with the average grain size of 18.3 μm. Its grain size distribution (Figure 1c) was well-fitted by the log-normal distribution function, which demonstrated the microstructure homogeneity in the initial material. In addition, there were abundant triple junctions with the grain boundaries angle of 120°, and this angle was generally considered to be an equilibrium angle [24]. Meanwhile, the grain boundaries at triple junction were relatively straight, indicating that current triple junctions were stable [24]. These results suggested that DRX was completed during the prior rolling process. In this work, the texture measurement of initial material was carried out on a JEOL 733 electron probe (JEOL, Tokyo, Japan) equipped with HKL Channel 5 system. Figure 1d shows the measured (0002), (10-10), and (11-20) pole figures of initial material. Clearly, the texture of initial material exhibited a similar (0002) basal texture, in which most c-axes were parallel to the normal direction. Its maximum texture intensity could achieve 10.25, and this was frequently seen in hot-rolled magnesium alloy sheets according to many studies [18,25].

The samples for microstructure characteristics were cut with the broad surface parallel to the RD–TD plane (RD: rolling direction, TD: transverse direction). After grinding with SiC paper and polishing with 0.05 μm silica suspension, these samples were etched with the use of a solution consisting of picric acid (5.5 g), acetic acid (2 mL), distilled water (10 mL), and ethanol (90 mL). Then, a OLYMPUS GX71 optical microscope (OM) (OLYMPUS, Tokyo, Japan) was applied for the metallographic observations. The mean linear intercept method was used to measure average grain size. However, according to the study by Chen [25], the relationship between the mean linear intercept ($\bar{L}$) and the spatial diameter ($\bar{d_{v}}$) is $\bar{d_{v}}=1.74\bar{L}$. Therefore, the average grain size obtained from the mean linear intercept method should be converted into the spatial diameters in the end, via multiplying the factor of 1.74. In addition, the twins in this work were not considered as grains, and did not participate in the calculation of average grain size. Apart from grain size, microstructure homogeneity was another key factor influencing the mechanical properties of magnesium alloys. Variance was an effective way to reflect the dispersion of sample mean. Therefore, it was here considered that the variance of grain size was used to characterize the microstructure homogeneity. In terms of texture measure, electron backscatter diffraction (EBSD) was conducted on the RD–TD plane of AZ31 magnesium alloy sheets. The preparation consisted of soft diamond polishing and subsequent etching with electropolishing in a 5:3 solution of ethanol and phosphoric acid for 8 min at 0.25 A. EBSD measurements were carried out on scanning electron microscopy (Quanta 200 FEG) (FEI, Hillsboro, AL, USA) equipped with EBSD system. An accelerating voltage of 20 keV, together with a working distance of 15 mm and a sample title angle of 70°, were selected to maximize the indexing of Kikuchi patterns. These EBSD data were analyzed using an Orientation Imaging Microscope (OIM) (FEI, Hillsboro, AL, USA). Using OIM software, the high angle grain boundary (HAGB) was identified according to the misorientation angle, whose values were above 15°. Uniaxial tension tests were performed on an Instron 5569 machine (Instron, Boston, MA, USA) with a strain rate of 1 × 10^−3^ s^−1^ at room temperature. In this work, the sheets rolled at 400 °C were used as the typical example to investigate the effect of thickness reduction on the mechanical properties of magnesium alloy sheets. Tension samples were cut by wire-cutting from the sheets rolled at 400 °C, along the rolling direction and the transverse direction. Their gauges were 25 mm in length and 6 mm in width. Three samples were each tested for thickness reduction, in order to ensure the repeatability of the results.

## 3. Results and Discussion

### 3.1. Microstructure Characteristics

**Figure 2:**
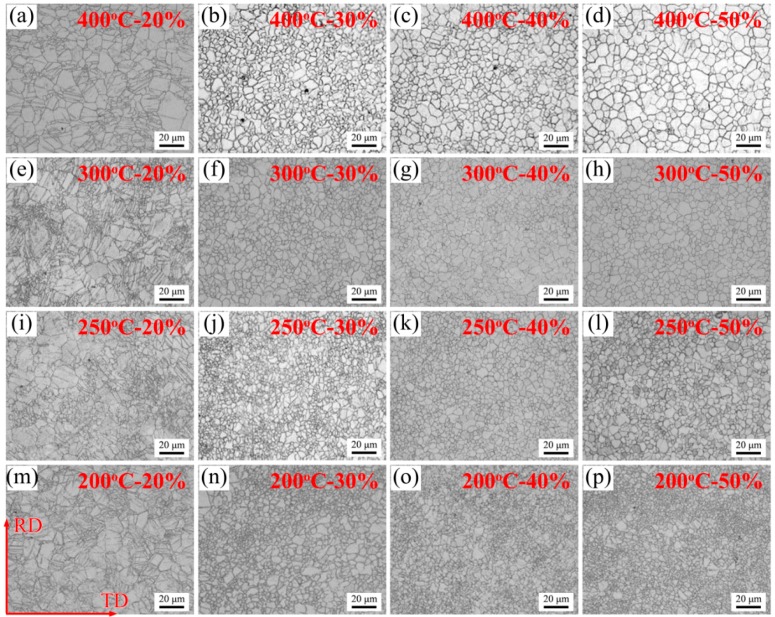
Metallographic observations of AZ31 magnesium alloy sheets under different rolling process parameters: (**a**) 400 °C—20%, (**b**) 400 °C—30%, (**c**) 400 °C—40%, (**d**) 400 °C—50%, (**e**) 300 °C—20%, (**f**) 300 °C—30%, (**g**) 300 °C—40%, (**h**) 300 °C—50%, (**i**) 250 °C—20%, (**j**) 250 °C—30%, (**k**) 250 °C—40%, (**l**) 250 °C—50%, (**m**) 200 °C—20%, (**n**) 200 °C—30%, (**o**) 200 °C—40% and (**p**) 200 °C—50%.

**Figure 3:**
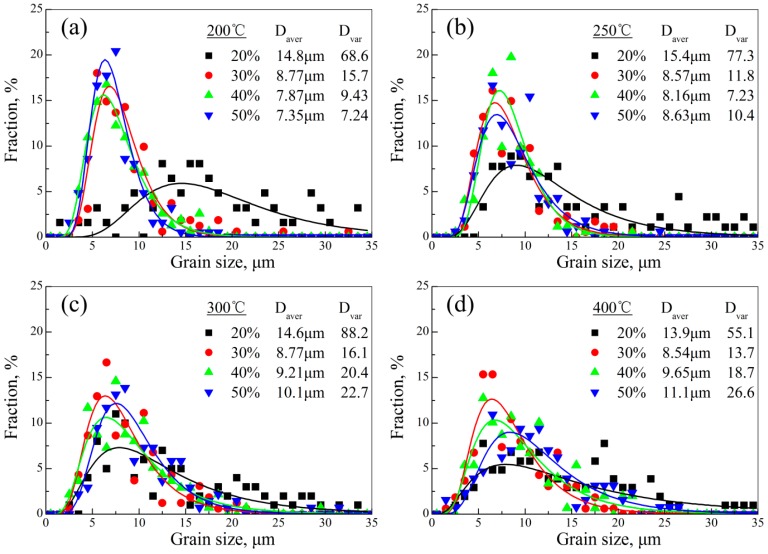
Grain size distributions of AZ31 magnesium alloy sheets under different rolling process parameters: (**a**) 200 °C, (**b**) 250 °C, (**c**) 300 °C, and (**d**) 400 °C.

Figure 2 shows the metallographic observations of AZ31 magnesium alloy sheets under different rolling process parameters. Their grain size distributions are summarized in Figure 3. Clearly, all the sheets after hot rolling possessed abundant twins when the thickness reduction was 20%. As the thickness reduction increased to 30%, these twins nearly disappeared and, meanwhile, a large number of fine equiaxed grains appeared, indicating that DRX behavior occurred at this stage. Apart from the 20% sheets, other grain size distributions in Figure 3 were well-fitted by the log-normal distribution function, which suggested the microstructure homogeneity. This was also a typical sign that DRX improved the microstructure homogeneity [26]. In addition, it was noteworthy that there existed remarkable differences in microstructure characteristics between 20% sheets and other sheets, i.e., the twin microstructure in 20% sheets and DRX microstructure in other sheets (30%, 40%, and 50%). This was mainly ascribed to the difference in deformation behavior during hot rolling. More specially, the above results demonstrated that the deformation was primarily accomplished via twins in 20% sheets, while this occurred via DRX in the other sheets (30%, 40%, and 50%). According to the literature [19,23], the critical deformation for twins was lower than that for DRX. If the amount of deformation was not attained in the critical deformation for DRX but for twins, then twin would be activated, which resulted in the release of stored energy and, then, the suppression of DRX. Figure 2 also shows that there existed a small quantity of DRXed grains in 20% sheets. Therefore, in the present work, the deformation was accommodated by twins in the majority, with a minor supplement of DRX at the amount of 20% deformation while, above 30%, the occurrence of DRX was the main mechanism for accommodating the deformation.

In terms of the effect of rolling temperature on DRX behavior, it played a similar role to the thickness reduction. Generally, lower deformation temperature led to increased critical deformation for DRX. This meant that DRX only occurred in some regions where the deformation was high enough, while not in other regions if the deformation was below the critical deformation, especially in the core of coarse grains. In view of this, it could be seen in the 200 °C—30% sheet (Figure 2n) that the DRXed fine grains only existed at the old grain boundaries, and many coarse grains still remained. This could be explained according to the fragmentation of coarse grain. Under the strain field of rolling process, the dislocations in coarse grain were rearranged into low angle grain boundaries (LAGBs). These LAGBs then progressively rotate, such that the misorientation increases continuously and, subsequently, HAGBs form. However, if the deformation was not enough, the fragmentation of coarse grain did not occur completely, which finally led to some remaining coarse grains [27]. Further observations on the 200 °C sheets could reveal that the fractions of these coarse grains decreased with the increase of thickness reduction. This could be ascribed to two factors: first, high thickness reduction played favor to increase the nucleation sits for DRX, so that the coarse grains were consumed more; second, high thickness reduction made more mechanical energy convert into heat energy of the sheets, which increased the deformation temperature and, subsequently, facilitated the occurrence of DRX.

**Figure 4:**
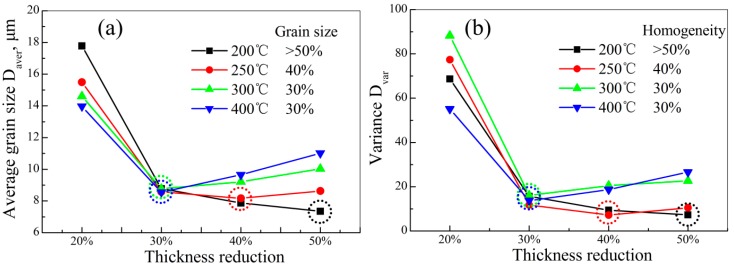
Average grain size variation (**a**) and corresponding variance of microstructure homogeneity of (**b**) AZ31 magnesium alloy sheets under different rolling process parameters.

As described above, DRX behavior was a comprehensive outcome of many factors, instead of depending on single factor. Microstructure controlling via DRX behavior was of importance in fabricating magnesium alloy sheets with high performance. This required a reasonable matching for rolling process parameters (thickness reduction and rolling temperature in this work). It is well known that fine grain size and homogeneous microstructure can improve the mechanical properties in magnesium alloys [5,28]. Thus, in this work, the grain size and microstructure homogeneity were regarded as the reference, whether the microstructure was good or not. Figure 4 shows the summarized results for average grain size variation and corresponding microstructure homogeneity (via variance) of AZ31 magnesium alloy sheets rolled under different rolling process parameters. Clearly, at a giving rolling temperature, the average grain size range was obviously refined to 8.54–8.77 μm in 30% sheets from 13.9–15.4 μm in 20% sheets. Then, with the increase of thickness reduction, this grain size refining effect was no longer significant. This was mainly ascribed to the deformation behavior, which was twinning in the 20% sheets, and DRX in the other sheets (30%, 40%, and 50%). Twinning refined the grain size by means of separating grains with twin boundaries while, for DRX, this was achieved by means of influencing the Zener–Hollomon parameter *Z* [29,30]. More critical was that, except in the case of the 200 °C sheets, the average grain size variation (Figure 4a) in other sheets had an inflection point, indicating that the average grain size would increase with the increase of thickness reduction. This could be attributed to that, on the premise of completed DRX, higher thickness reduction increased the rolling temperature of the sheet (as described before), which was conducive to the growth of DRXed grain. However, as for 200 °C sheets, no inflection point appeared. It could be predicted that the grain would be further refined if the thickness reduction continued to increase. Clearly, the inflection points were 400 °C—30%, 300 °C—30%, and 250 °C—40% (Figure 4a). In other words, this matching of rolling temperature and thickness reduction was the critical rolling process parameter for fabricating AZ31 magnesium alloy sheets with fine grain size. With regard to the microstructure homogeneity, the variance variation was similar to the average grain size, and had same inflection points. This suggested that the above matching of rolling process parameters was also conducive to realizing the microstructure homogeneity. Therefore, if grain size and microstructure homogeneity were used as the reference, whether the microstructure was good or not, the critical rolling process parameters were 400 °C—30%, 300 °C—30%, and 250 °C—40% for AZ31 magnesium alloy sheets.

**Figure 5:**
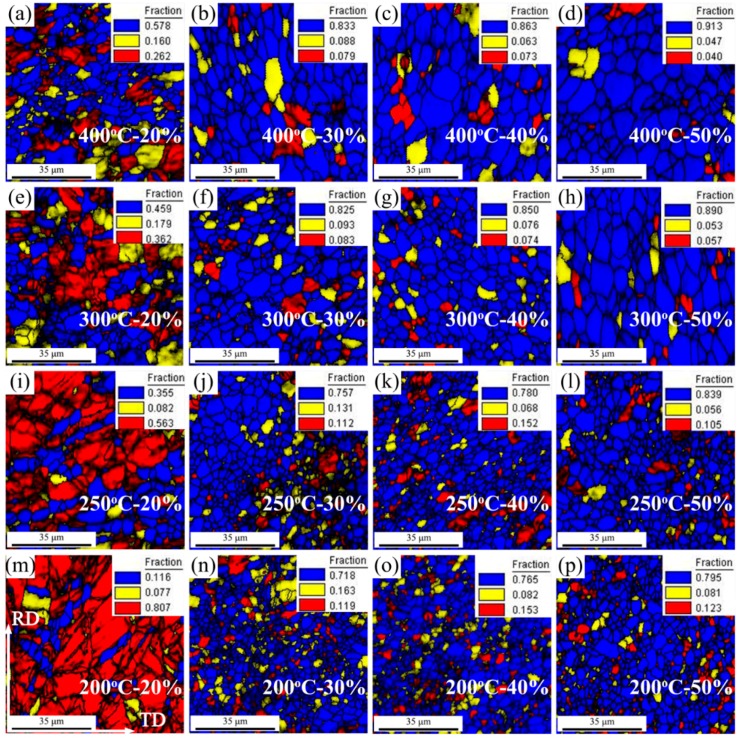
Microstructure characteristic maps of DRXed grains, subgrains, and deformed grains of AZ31 magnesium alloy sheets under different rolling process parameters: (**a**) 400 °C—20%, (**b**) 400 °C—30%, (**c**) 400 °C—40%, (**d**) 400 °C—50%, (**e**) 300 °C—20%, (**f**) 300 °C—30%, (**g**) 300 °C—40%, (**h**) 300 °C—50%, (**i**) 250 °C—20%, (**j**) 250 °C—30%, (**k**) 250 °C—40%, (**l**) 250 °C—50%, (**m**) 200 °C—20%, (**n**) 200 °C—30%, (**o**) 200 °C—40% and (**p**) 200 °C—50% (recrystallized grain—blue region; subgrain—yellow region; deformed grain—red region).

**Figure 6:**
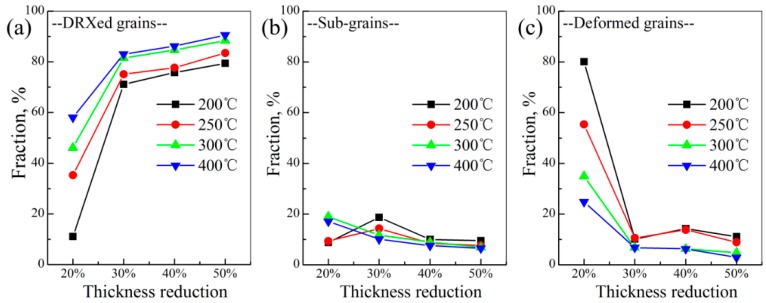
Fraction variation of DRXed grains (**a**), subgrains (**b**), and deformed grains (**c**) under different rolling process parameters.

In the Orientation Imaging Microscope (OIM) software, the grain average misorientation (GAM) was determined by calculating the misorientation between each neighboring pair of points within the grain [10]. This was frequently used to reflect the dislocation density and further characterize recrystallized grains, subgrains, and deformed grains, by setting the GAM values [10,11]. In this work, DRXed grains were identified by GAM values smaller than 1.2° (blue region), subgrains between 1.2° and 2.5° (yellow region), and deformed grains greater than 2.5° (red region). These values were selected according to the literature [10]. In this work, the EBSD measurement area was selected randomly in the samples, and the size was 78 μm × 78 μm. The recrystallization fraction was calculated based on more than 150 grains. Based on the above method, the microstructure characteristics of DRXed grains, subgrains, and deformed grains of AZ31 magnesium alloy sheets, under different rolling process parameters, are shown in Figure 5. Their corresponding fractions are summarized in Figure 6. Overall, higher rolling temperature and higher thickness reduction was beneficial to increase the fractions of lower GAM value region (blue region), clearly seen in Figure 5 and Figure 6. Specially, there still existed a significant difference in the fraction of blue region between the 20% sheets and other thickness reduction sheets: a maximum value of only 57.8% was observed in 20% sheets, while the minimum in other sheets surpassed 71.8%. This was mainly ascribed to the difference in the behavior of accommodating the deformation. As described above, all the 20% sheets relied on twinning to accommodate the deformation and, in this case, the dislocation could not be eliminated, which resulted in a larger deformed region (red region) in the 20% sheets. However, for other thickness reduction sheets, the occurrence of DRX produced many new grains with low dislocation density, which made the fraction of lower GAM value region (DRXed grain: blue region) increase significantly. For deformed grains (red region), their fraction exhibited a corresponding trend, clearly seen in Figure 6c.

In addition, two points should be noted. First, for the 20% sheets, the fraction of lower GAM value region (blue region) increased significantly with the increase of rolling temperature (11.6% at 200 °C; 57.8% at 400 °C). Under this thickness reduction, although DRX rarely occurs, dynamic recovery still appeared, which would reduce the dislocation density by dislocation climb, cross-slip, and glide. Moreover, this dynamic recovery generally occurred more at higher deformation temperature. Therefore, the 400 °C—20% sheet exhibited a larger fraction of lower GAM region (blue region). Second, in the sheets with DRX behavior (thickness reduction >30%), the fraction of lower GAM region (DRXed grain) increased with the increase of thickness reduction. This could be attributed to two factors. First, higher thickness reduction produced more nucleation sites, which realized completed DRX more easily; second, higher thickness reduction made new DRXed grains with low dislocation density grow more easily (as described before).

### 3.2. Texture Characteristics

As mentioned earlier, under the rolling process parameters of 400 °C—30%, 300 °C—30%, and 250 °C—40%, AZ31 magnesium alloy sheets possessed optimal grain size and microstructure homogeneity. However, for magnesium alloys, their HCP structure was liable to cause strong basal texture, which greatly influenced the mechanical properties [28,31]. During the hot rolling process, both higher deformation temperature and higher thickness reduction could influence the texture state of magnesium alloy sheets. The former promoted the non-basal slip to be activated by lowering their critical resolved shear stress (CRSS), while the latter increased the stress concentration which would also activate the non-basal slip. More importantly, DRXed grains and their growth in the hot rolling process would participate in texture evolution and this, finally, influenced the texture state of the resulting magnesium alloy sheets [9,19]. Therefore, it was necessary to investigate the influence of rolling process parameters on the texture state in the resulting magnesium alloy sheets, especially when DRX occurred. In this work, 400 °C sheets were used as the typical example to investigate the effect of thickness reduction on the texture of magnesium alloy sheets. The reason for selecting these sheets was that, at 400 °C, DRX occurred easily in AZ31 magnesium alloy sheets, and grew distinctly with the increase of thickness reduction.

**Figure 7:**
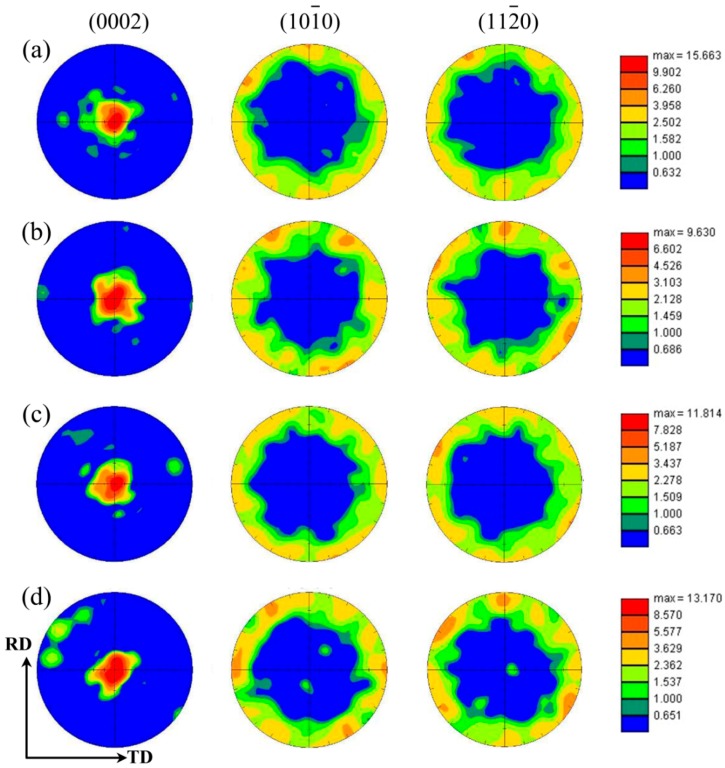
(0002), (10-10), and (11-20) pole figures of AZ31 magnesium alloy sheets for (**a**) 400 °C—20%, (**b**) 400 °C—30%, (**c**) 400 °C—40%, and (**d**) 400 °C—50% (RD: rolling direction, TD: transverse direction).

Figure 7 shows the (0002), (10-10), and (11-20) pole figures of 400 °C sheets with different thickness reduction. Clearly, all the sheets exhibited a strong basal texture, in which nearly all the c-axes were parallel to the normal direction (ND). This was frequently seen in rolled sheets, and its formation could be attributed to the stress state in rolling process [16,32]. According to the literature [33], the slip plane would gradually rotate toward the rolling plane. Since basal slip was the main deformation mode in magnesium alloys, due to its lower CRSS [34], they would gradually rotate toward the rolling plane under the combined effect of compression stress along the ND, and tension stress along the RD. Moreover, many studies had demonstrated that the occurrence of DRX mainly affected the rotation of crystal orientation, without causing the deflection of basal plane [9,27,33]. In other words, the final strong basal texture formed in the hot rolling process was primarily dependent on the texture state after deformation. Of course, if the dominant deformation mechanism was twin, the lattice rotation was complicated because of the manifold twin systems which would induce different grain boundary misorientation, such as 86° for the (10-12) tension twin, and 38° for the (10-11)–(10-12) double twin [35]. However, tension twin was frequently observed, owing to its lower CRSS compared to other twin systems [29]. Therefore, the twin in 20% sheets was roughly deemed to be (10-12) twin in this work.

**Figure 8:**
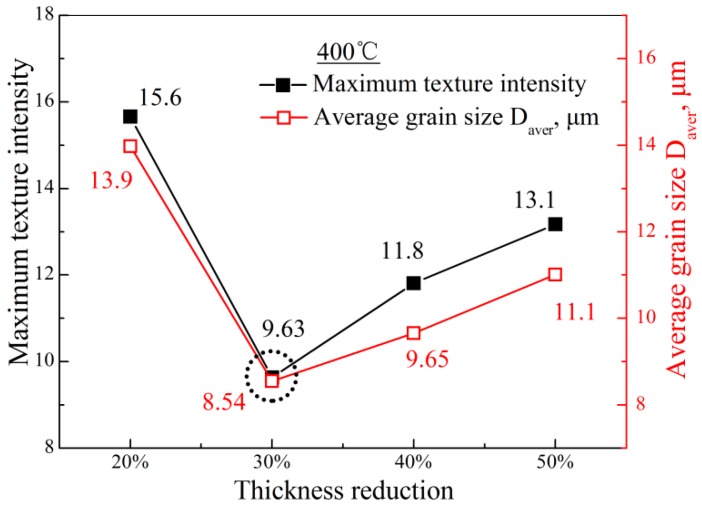
Maximum texture intensity variation and corresponding average grain size variation with the increase of thickness reduction at a given rolling temperature of 400 °C.

Figure 7 displays obvious differences of maximum texture intensity in the four thickness reduction sheets. In order to clearly assess this difference, their maximum texture intensities are summarized in Figure 8. As shown, the maximum texture intensity was the largest in 400 °C—20% sheet (15.6), and then sharply decreased to 9.63 in the 400 °C—30% sheet. After a thickness reduction of 30%, the maximum texture intensity steadily increased with the increase of thickness reduction. These phenomena should be explained separately. In terms of the sharp decrease from 20% to 30%, it was attributed to the competition between DRX and twin. As mentioned earlier, in the 20% sheet, tension twin was the main deformation mode, and this would induce a lattice reorientation of 86°, which would rotate the basal plane toward the rolling plane rapidly [36]. However, for the sheets with DRX behavior, dislocation slip dominated the plastic deformation and subsequent DRX. It is well known that the lattice rotation induced by dislocation slip was a gradual process, which made the basal plane not rotate as quickly as tension twin. Therefore, the maximum texture intensity would sharply decrease to 9.63 when DRX occurred in the 400 °C—30% sheet. In terms of the variation from 30% to 50%, it was most likely due to the growth of DRXed grain, which was judged according to the result in Figure 8, that the maximum texture intensity variation was similar to the average grain size with the increase of thickness reduction. Imandoust et al. once reported that, compared to the DRXed grains, the texture intensity in DRXed grains bigger than 25 μm obviously increased, and their spread was more concentrated [8]. Subsequently, they analyzed this observation by migratory direction of high angle grain boundary [8]. They pointed out that the grains with lower Schmid factor generally had lower dislocation density, and this produced a dislocation density gradient, which applied a force to the boundary toward the grain with higher dislocation density [8]. Finally, the texture of the grains with lower dislocation density would be the dominant texture component with these grains’ growth [8]. In this work, higher thickness reduction generally increased the deformation temperature and, then, made DRXed grains (lower dislocation density grains) coarsen. These coarsened DRXed grains would gradually invade and occupy other regions with their growth, which made their texture characteristics more obvious in coarse grain microstructure. Figure 8 also indicated that at the rolling temperature of 400 °C—30% was the optimal thickness reduction if texture weakening was used as the reference.

**Figure 9:**
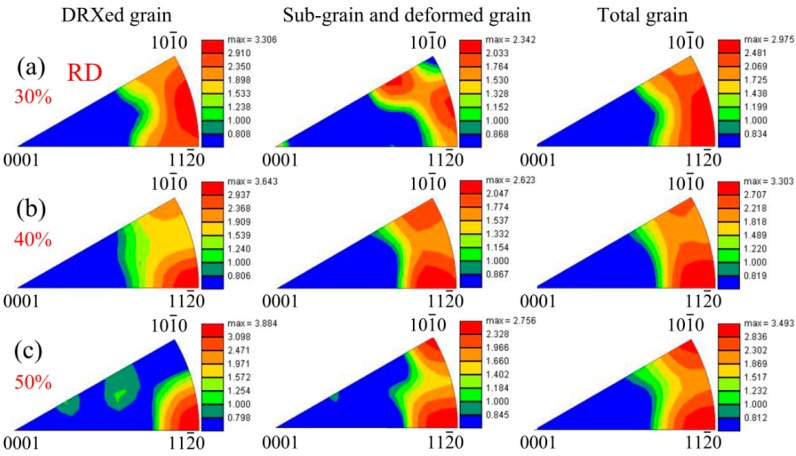
Inverse pole figures along the RD for dynamic recrystallized (DRXed) grain region, subgrain, and deformed grain region, and total grain region in (**a**) 400 °C—30%, (**b**) 400 °C—40%, and (**c**) 400 °C—50%.

Generally speaking, the microstructure after hot rolling process could be divided into DRXed grains, subgrains, and deformed grains, and according to many studies, their texture states were different, especially the crystal orientation along the RD [5,9]. In order to contrast this difference, their inverse pole figures along the RD were obtained according to the result in Figure 5. Here, the DRXed grain region, subgrain and deformed grain region, and total grain region were observed, which is shown in Figure 9. As shown, the texture in DRXed grain region had a trend of becoming <11-20>//RD texture with the increase of thickness reduction. Barrett et al. analyzed the texture state of parent grain (deformed grain) and DRXed grain in extruded AM30 magnesium alloy, and found that the latter had two types of fiber texture: a newly preponderant <11-20> fiber texture component and an inborn <10-10> fiber texture component [9]. As in a static recrystallization process, the DRXed grains with <11-20> fiber texture component had lower Schmid factor and lower dislocation density [8]. As mentioned above, these DRXed grains grew so easily that their texture component would become the dominant texture component, while the grains with <10-10> fiber texture component would gradually disappear. In this work, DRXed grains coarsened with the increase of thickness reduction. This meant that the DRXed grain with <11-20>//RD texture component would gradually annex the <10-10>//RD grain regions with the increase of thickness reduction. As a result, the <11-20>//RD texture component became dominant texture state in the 400 °C—50% sheet. For subgrain and deformed grain regions, <10-10>//RD texture and <11-20>//RD texture looked coexistent. This could be ascribed to the combining effect of subgrains and deformed grains. More specifically, the subgrains would inherit some characteristics of <11-20>//RD texture arising from DRXed grain, despite producing a slight transformation as the deformation proceeded. For deformed grains, many studies have demonstrated that their <10-10> would gradually rotate toward the RD [5,6,27]. Therefore, by superposition of the texture components above, the subgrain and deformed grain region exhibited the coexistence of <10-10>//RD texture and <11-20>//RD texture. However, when the deformation continued, DRXed grains would experience plastic deformation again, which made their <10-10> rotate toward the RD again. This meant that the crystal orientation in total grain region spread along <10-10>–<11-20> arc in the inverse pole figure, especially for the texture state in the total grain region of 400 °C—30% sheet (Figure 9a).

### 3.3. Tensile Test Results

**Figure 10:**
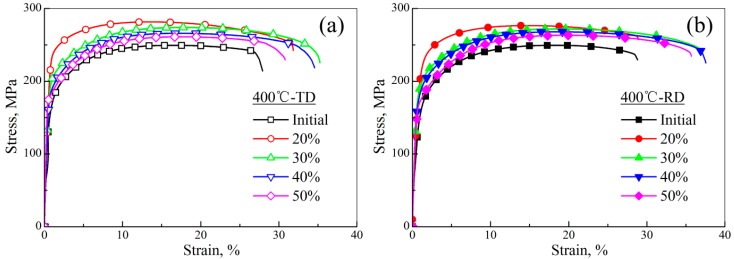
Stress–strain curves of AZ31 magnesium alloy sheets rolled at 400 °C: (**a**) TD; (**b**) RD.

**Figure 11:**
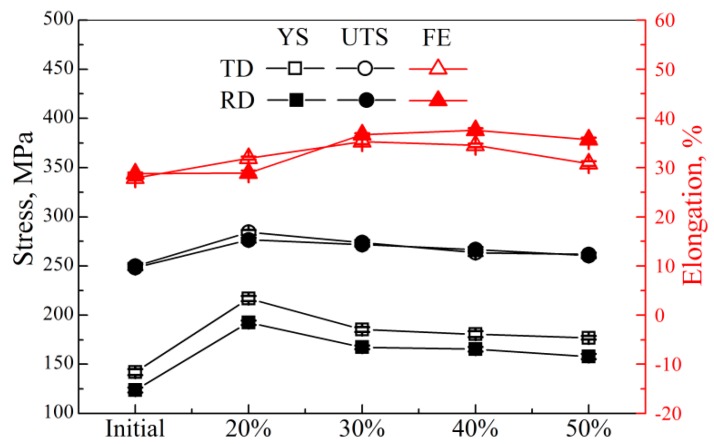
Stress variation and fracture elongation variation with the increase of thickness reduction at a given rolling temperature of 400 °C (YS: yield stress, UTS: ultimate tension stress, FE: fracture elongation).

Figure 10 shows the stress–strain curves of AZ31 magnesium alloy sheets at a given rolling temperature of 400 °C. Their yield stress (YS, MPa), ultimate tension stress (UTS, MPa), and fracture elongation (FE, %) are summarized in Table 2, and their corresponding variations are shown in Figure 11. Clearly, the YS along both directions in resulting sheets increased obviously, compared to the initial material, which could be ascribed to the contribution of grain refinement to yield stress based on the Hall–Petch relationship [37]. Moreover, the yield stress in 20% sheets was distinctly higher than other sheets. As described above, 20% sheets possessed massive deformed grains. In other words, strain hardening was more obvious in these sheets and, finally, led to their higher yield stress. However, this work focused on the elongation of magnesium alloys. From Figure 11, 30% and 40% sheets had the highest fracture elongation (36.7% in 30% sheets, and 37.6% in 40% sheets along the RD; 35.3% in 30% sheets, and 34.5% in 40% sheets along the TD). Generally, the fracture elongation could be divided into two parts: uniform elongation and post-uniform elongation. According to Chen’s study, weak basal texture contributed to the uniform elongation, while grain refinement contributed to the post-uniform elongation [25]. As mentioned above, for 400 °C sheets, 30% and 40% sheets possessed the smaller grain size (30%: 8.54 μm; 40%: 9.65 μm) and the smaller maximum texture intensity (30%: 9.63; 40%: 11.8). Therefore, 30% and 40% sheet had higher fracture elongation. In other words, when the rolling temperature was 400 °C, a 30–40% thickness reduction was conducive to fabricating AZ31 magnesium alloy sheets with better fracture elongation, and could be the critical thickness reduction.

## 4. Conclusions

In this work, the DRX behavior of AZ31 magnesium alloy sheets was investigated under various rolling temperatures and thickness reductions. Results indicated that reasonable matching of rolling process parameters was conducive to realizing the grain refinement and improving microstructure homogeneity. According to the grain size and microstructure homogeneity, the critical rolling process parameters were 400 °C—30%, 300 °C—30%, and 250 °C—40% for AZ31 magnesium alloy sheets. In terms of the basal texture variation, with the increase of thickness reduction, the competition between twinning and DRX resulted in an obvious difference in maximum texture intensity. The former produced the largest maximum texture intensity in 400 °C—20% sheets. However, due to the occurrence of DRX, the maximum texture intensity decreased sharply, but would steadily increase with the growth of DRXed grain. In addition, for DRXed grains, <11-20>//RD texture component would gradually become the dominant texture state, owing to their growth. However, when the deformation continued, <10-10> would rotate toward the RD again. From the fracture elongation perspective, the critical thickness reductions of AZ31 magnesium alloy sheet were 30–40% when the rolling temperature was 400 °C.

## Figures and Tables

**Table 1:** Rolling process parameters.

Initial Thickness (mm)	Rolling Temperature (°C)	Thickness Reduction (%)			
4	400	20	30	40	50
4	300	20	30	40	50
4	250	20	30	40	50
4	200	20	30	40	50

**Table 2:** Summarized mechanical properties of AZ31 magnesium alloy sheets rolled at 400 °C (YS: yield stress; UTS: ultimate tension stress; FE: fracture elongation).

Condition	YS, MPa		UTS, MPa		FE, %	
	TD	RD	TD	RD	TD	RD
Initial	142.1 ± 3.1	123.8 ± 2.6	249.7 ± 3.3	248.5 ± 2.9	27.9 ± 0.5	28.8 ± 0.2
20%	216.8 ± 2.8	192.3 ± 2.3	284.1 ± 2.6	276.3 ± 2.4	31.9 ± 0.3	28.9 ± 0.5
30%	185.3 ± 2.3	167.3 ± 1.8	273.9 ± 2.4	271.8 ± 2.0	35.3 ± 0.6	36.7 ± 0.3
40%	180.6 ± 3.2	165.4 ± 2.4	263.5 ± 3.3	266.5 ± 2.7	34.5 ± 0.4	37.6 ± 0.4
50%	177.0 ± 1.9	157.9 ± 2.7	261.4 ± 2.2	260.7 ± 2.8	30.8 ± 0.5	35.7 ± 0.4
